# Metformin as a new option in the medical management of breast fibroadenoma; a randomized clinical trial

**DOI:** 10.1186/s12902-021-00824-4

**Published:** 2021-08-20

**Authors:** Sadaf Alipour, Mahboubeh Abedi, Azin Saberi, Arezoo Maleki-Hajiagha, Firoozeh Faiz, Saeed Shahsavari, Bita Eslami

**Affiliations:** 1 Breast Disease Research Center, Cancer Institute, Tehran University of Medical Sciences, Tehran, Iran; 2 Department of Surgery, Arash Women’s Hospital, Tehran University of Medical Sciences, Tehran, Iran; 3 Department of Radiology, Arash Women’s Hospital, Tehran University of Medical Sciences, Tehran, Iran; 4 Research Development Center, Arash Women’s Hospital, Tehran University of Medical Sciences, Tehran, Iran; 5 Department of Endocrinology and Metabolism, Arash Women’s Hospital, Tehran University of Medical Sciences, Tehran, Iran; 6 Department of Epidemiology and Biostatistics, School of Public Health, Tehran University of Medical Sciences, Tehran, Iran; 7 Health Products Safety Research Center, Qazvin University of Medical Sciences, Qazvin, Iran

**Keywords:** Fibroadenoma, Fibrocystic Breast Disease, Breast Ultrasonography, Metformin, Therapy

## Abstract

**Background:**

Fibroadenoma (FA) is the most common benign solid breast mass in women, with no definite method of management. Because fibroadenoma is dependent on female sex hormones and comprises hypertrophic changes at cellular levels, we investigated the effects of metformin (MF), a safe hypoglycemic agent with anti-estrogenic and anti-proliferative properties, in the management of fibroadenoma.

**Methods:**

In this randomized clinical trial study, eligible women with fibroadenomas were assigned randomly to the metformin (1000 mg daily for six months) or the placebo group. Breast physical and ultrasound exam was performed before and after the intervention, and the changes in the size of fibroadenomas were compared in the two groups.

**Results:**

Overall, 83 patients in the treatment, and 92 in the placebo group completed the study. A statistically significant difference in changing size between the two groups was observed only in the smallest mass. In the largest FAs, the rate of size reduction was higher in the treatment group (60.2 % vs. 43.5 %); while a higher rate of enlargement was observed in the placebo group (38 % vs. 20.5 %). In the smallest FAs, the rate of the masses that got smaller or remained stable was about 90 % in the treatment group and 50 % in the placebo group. We categorized size changes of FAs into < 20 % enlargement and ≥ 20 % enlargement. The odds ratio (OR) for an elargemnt less than 20% was 1.48 (95 % CI = 1.10–1.99) in the treatment group in comparison with the placebo group; the odds for an enlargement less than 20% was higher in women with multiples fibroadenomas (OR = 4.67, 95 % CI: 1.34–16.28). In our study, no serious adverse effect was recorded, and the medicine was well-tolerated by all users.

**Conclusions:**

This is the first study that evaluates the effect of MF on the management of fibroadenoma, and the results suggest a favorable effect. Larger studies using higher doses of MF and including a separate design for patients with single or multiple FAs are suggested in order to confirm this effect.

**Trial registration:**

This trial (IRCT20100706004329N7) was retrospectively registered on 2018-10-07.

## Background

Fibroadenoma (FA) is the most common benign solid mass of the female breast, with an approximate incidence rate of around 12–25 % in young women, albeit the exact incidence is not known. It is most commonly seen in young women between 14 and 35 years old and is much less common in post-menopausal women, but can occur at any age [1, 2]. FA can present as a solitary mass in one breast, or as multiple bilateral lumps, and can sometimes grow to very large sizes. Palpable FA has a typical appearance consisting of a firm, round, very mobile lump; however, in many instances, FA is not palpable and can only be detected by breast imaging. The typical ultrasound (US) picture is a circumscribed, regular, hypoechoic mass that lies parallel to the skin. Both clinical and paraclinical presentations can be atypical and different from the usual image [1, 2].

The main underlying etiology is unknown, but the similarity of the effects of sex hormones on FA and normal breast tissue suggests a hormonal pathophysiology [2]. Mild stromal and epithelial proliferative changes are seen in FA histology [3, 4]. Women with FA are at a slightly increased risk of developing malignancy in comparison with the general population [5]. Diagnosis is based on histological examination which is available through core needle biopsy of the lesion. Cytological assessment also is helpful but not as accurate. Nonetheless, for a small FA with typical features on physical exam or US scan in a young woman, tissue sampling can be avoided; and the diagnosis can be made clinically with relative accuracy in these cases [1].

Despite the relative benignity of FA, it can impose a significant negative psychological impact on the patient. Stress about misdiagnosis, probable malignant transformation, or even feeling of fear while touching the lump are not uncommon consequences of conservative treatment [2].

Metformin (MF) is an anti-hyperglycemic agent that is being investigated for many medical disorders and conditions. One of the probable properties of MF is its anti-proliferative effects on various cells, including breast cancer cells. Also, anti-estrogenic properties have been reported for MF [6]. Because of the estrogen-dependent and proliferative features of FA [2], and the anti-proliferative, sex hormone-suppressing characteristics of MF [6], as well as its relatively low frequency of adverse effects, we designed the present study to evaluate the therapeutic effects of MF on FA.

## Methods

### Study design and participants

 This study was conducted according to the principles of the Declaration of Helsinki and the standards of Good Clinical Practice (GCP); and has been approved by the Institutional Research Board (Proposal Code: 97-01-218-37716) and the Ethics Committee (Approval ID: IR.TUMS.VCR.1397.357) of Tehran University of Medical Sciences, Tehran, Iran. This study adheres to CONSORT guidelines. It has been retrospectively registered in the Iranian Registry of Clinical Trials (IRCT), registration number: IRCT20100706004329N7. This is a Primary Registry in the WHO Registry Network set up by the Ministry of Health and Medical Education (MOHME).

This is a single-center, double-blind, randomized placebo-controlled clinical trial with a parallel-group design that has been held in Arash Women’s Hospital, affiliated to Tehran University of Medical Sciences from October 2018 to March 2020. The study population consisted of women attending the breast clinic of the hospital. All participants read and signed a written informed consent before entering the study.

### Study Outcomes

The primary outcomes (and the relevant anticipated favorable results) consisted of the following:

1. Change in size of the largest lesion (a less than 20 % increase in a 6-month interval was considered as favorable).
2. Change in number of detectable lesions (an overall decrease was considered as favorable).
3. Change in average size of all lesions (a less than 20 % increase in a 6-month interval for any FA, and in multifocal cases a less than 20 % enlargement in all the masses of a patient was considered as favorable ).

The secondary outcomes consisted of the occurrence of drug adverse effects and compliance with regular consumption of the medication.

### Inclusion criteria

Premenopausal women aged 18–50 years old with one or more, unilateral or bilateral FA less than 3 cm in largest diameter were included in the study. Criteria for the diagnosis of FA were the criteria we usually use in our clinic based on the largest diameter of the lump on US scan or breast examination.

-In women younger than 40 years of age:

*For lumps less than 2 cm: typical US image of FA, and typical physical exam when palpable.

*For lumps 2 cm or larger: a diagnosis of FA in histologic exam of core needle biopsy samples.

- In women 40 years of age or above:

*For lumps larger than 1 cm: a diagnosis of FA in histologic exam of core needle biopsy samples.

*For lumps less than 1 cm in women with no risk factor for breast cancer and no suspicious finding in mammography: a typical US image.

*For lumps less than 1 cm in women with a risk factor for breast cancer or a suspicious image in mammography: FA in histologic exam of core needle biopsy samples.

- In all ages:

*For multiple lumps that have been stable for one year or more: a diagnosis of FA in histologic exam of core needle biopsy samples of only the largest one and/or those above 2 cm.

*For any lump based on patient or clinician preference.

### Exclusion criteria

These consisted of pregnancy, breastfeeding, vegetarianism, body mass index (BMI) more than 29.9, history of breast cancer, allergy to biguanides, present diabetes mellitus, hypothyroidism, hyperthyroidism, metabolic syndrome, galactorrhea, hypophysis adenoma, heart disease, epilepsy, renal or hepatic failure, severe iron deficiency anemia, gastroparesis, or severe hyperlipidemia; use of anti-diabetics and hypoglycemic agents, antilipidemics, phytoestrogen containing medications, GnRH agonists and antagonists, clomiphene, tamoxifen, aromatase inhibitors, danazol, oral contraceptives or any medicine containing estrogens or progestins or the history of using these products during the last two years; getting pregnant during the study, showing adverse effects of MF, irregular use of the medication or complete non-compliance.

### Random Allocation, Concealment, and Blinding

Random allocation was performed by a methodologist using an online generated randomization list (provided by sealedenvelope.com) based on the block randomization method and 6-piece blocks. The randomization list was concealed from all research staff involved in the enrollment and assessment of patients by using sealed envelopes. For blinding, the MF and identical placebo tablets were placed in similar bottles with similar labels. Then, bottles were stored in two separate boxes that were coded as A or B by individuals who were not involved in drug dispensing, patient visit, and follow-up. Coding of A and B were defined and kept in an envelope, which was disclosed after the analysis of the results. Participants were allocated to group A or B according to the randomization list, and then the medication dispenser provided the participants with the drug bottle from the corresponding box.

### Interventions, measurements, and tests

Eligible women were enrolled in the study by surgeons of the breast clinic based on US findings and/or histology results. All participants underwent a physical examination and breast US. All women aged 40 years and older had undergone mammography in the recent year. US scans were performed by a radiologist experienced in breast US and dedicated to the breast clinic.

Every participant filled in a form containing questions about demographic information, previous breast disease, and personal, menstrual, reproductive, and past medical information. Height, weight, waist and hip circumference of all participants were measured by one trained personnel.

 Blood tests including complete blood count, blood sugar, liver and renal function tests were performed for all participants. People with abnormal results were excluded from the study and referred for appropriate management. Then, participants were allocated into treatment and placebo groups. The treatment group received standard-release tablets of 500 milligrams MF (Osveh Pharmaceutical Company, Iran) twice daily for six months. The placebo group received placebo tablets that were quite similar to MF tablets (Osveh Pharmaceutical Company, Iran) twice daily. Women in both groups were asked not to change their routine dietary habits. They were also requested to inform their doctors about any changes in their diet and medications, or newly diagnosed diseases.

A drug-reminder table was given to each participating woman to check, record, and trigger their compliance with the assigned intervention and checkmark the corresponding box each time they consumed the tablet. They were also given only one drug bottle containing tablets for three months of use and were asked to attend three months later for the second box. Also, short messages were sent every two weeks as a reminder to use the drugs regularly, and they were asked to come for the second drug bottle by phone call. The second US scan and the last examination were scheduled by phone calls. At the end of the sixth month of intervention, the breast US scan, anthropometric measurements, and renal function test were repeated for all participants who had fulfilled the intervention and the size of FA and results of measurements were recorded.

### Sample size calculation

Since this study evaluates the effect of MF on FA for the first time, we used the study of Tejwani et al., [7] who prescribed centchroman in fibroadenomas, for calculating the sample size. While the size decrement in that study was 19 % in the control arm and around 52 % in the case group, by considering a power of 90 % and α = 0.05, a sample size of 48 patients was needed in each group. However, due to our experts opinion that predicted a lower effect for metformin (because centchroman is an estrogen receptor modulator), we planned to gather a sample size of 100 participants in each group.

### Statistical Analysis Methods

The statistical analyses were performed using IBM SPSS 24 (IBM Corp. Released in 2016. IBM SPSS Statistics for Windows, Version 24.0. Armonk, NY: IBM Corp). Data are presented as mean ± standard deviation for continuous variables and number with percentages for categorical variables. Comparison between the two groups was conducted by Student t-test and Chi-square test. Comparison between pre and post-intervention FA size in each group was conducted using Paired-t-test.

We evaluated the results from two perspectives: In terms of changes of mass size and changes of mass number. We evaluated the changes in size of the largest and smallest masses in each woman as a continuous variable. Then we categorized changes in the size of FA after the intervention compared with their basal size into three groups: no change, enlargement, and size decrement. For women with a single mass, that mass was considered as the largest. The smallest mass was considered only in women who had multiple masses. The total number of breast masses and average mass size before and after the intervention were calculated in each woman and the differences were compared between two groups. In order to analyze the changes in mass number, we categorized the changes in three groups including disappearance of all FA, reduction in the number of FAs and no change, and increase in the number of FAs.

Considering that one of the main clinical concerns about FA is size stability, and since a 20 % enlargement is contemplated as significant [8–10], we categorized size changes for each FA as < 20 % enlargement (including also size reduction and size stability), and ≥ 20 % enlargement. In patients with multiple FAs, since medical management of FA cannot target every single mass, we considered < 20 % FA enlargement –encompassing size stability or regression- in all the masses of a patient as favorable, and ≥ 20 % FA enlargement in even one mass of a patient as unfavorable. The percentage of change of the FA size was calculated as the ratio of mass size before minus after intervention over before intervention, or (Size after – Size before / Size before) × 100.

Since there was more than one mass in many of the participants, we realized that we could not consider each mass separately, because FAs that were present in the breast of one woman were correlated to each other. Therefore by contemplating the fact that our quantitative response data were correlated, marginal model and generalized estimate equation (GEE) model with exchangeable correlation matrix was performed for comparison of changes. GEE is a quasi-likelihood approach for correlated data which does not fully specify the distribution of response in each patient as a cluster [11]. The comparison between study groups was performed in the GEE model, where more than one measurement of each patient was treated as correlated, baseline measurements were considered as covariates and the study group as the independent effect factor. In addition, we used logistic regression when our response was binary. A p-value of less than 0.05 was considered significant. Moreover, all of the dropped-out cases occurred before the first follow-up visits. Therefore, we had no intention-to-treat analysis.

## Results

### Flow of Patients

**Fig. 1:**
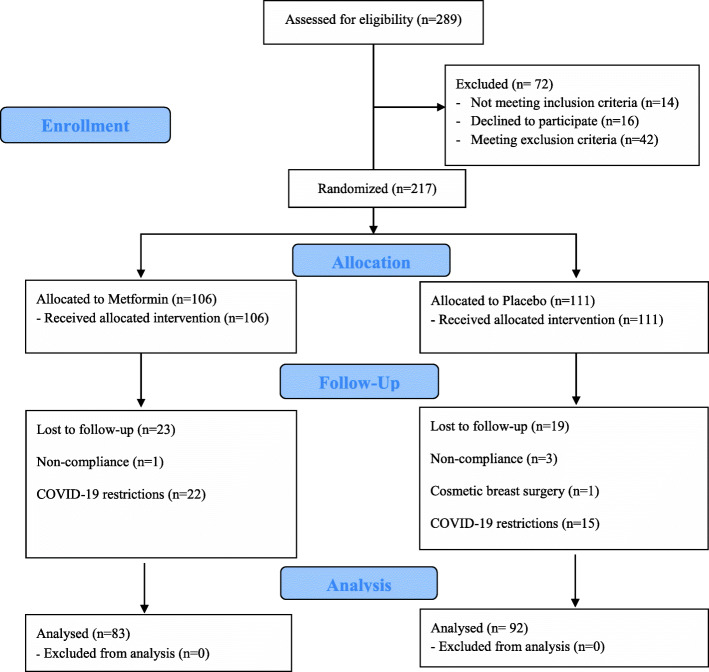
Summary of CONSORT flowchart

First, 217 patients were enrolled in the study, consisting of 111 women in the placebo group and 106 patients in the treatment group. Four women (one in the treatment and three in the placebo group) were excluded during the intervention due to incompetency with drug adherence, and one in the placebo group underwent cosmetic reduction mammoplasty and was withdrawn. Also, COVID-19 restrictive conditions supervened throughout the study, thus 22 and 15 patients were lost to follow-up in the treatment and placebo groups, respectively. Ultimately, 175 patients completed the study; 83 in the treatment and 92 in the placebo group. These are demonstrated in Fig. 1.

### Characteristics of Patients and Fibroadenomas

The mean age of all the participants was 39.65 ± 10.30 years. The two groups were similar regarding age, BMI and waist to hip ratio. Demographic, anthropometric, and reproductive characteristics of patients in the two groups at the time of entering the study are demonstrated in Table 1. Multiple FAs were seen in 80 patients (45.7 %), and 95 women (54.3 %) had a single FA. Considering diagnostic criteria for inclusion, biopsy and histologic diagnosis of FA had been done for 74 patients with single FA (75.5 %), and for 68 patients with multiple FAs (88.3 %).

**Table 1:** Demographic, anthropometric, and reproductive features in the two groups at the time of entering the study

Variables	Metformin Group (*n* = 83)	Placebo Group (*n* = 92)	*p*-value
**Age**	39.90 ± 10.54	39.42 ± 10.14	0.76^a^
**Body mass Index (BMI)**	25.51 ± 4.72	26.36 ± 6.95	0.35^a^
**Waist/hip ratio**	0.86 ± 0.07	0.86 ± 0.13	0.75^a^
**Age of menarche**	13.13 ± 1.33	13.32 ± 1.39	0.37^a^
**Parity**	1.53 ± 1.13	1.51 ± 1.10	0.91^a^
**Age at first delivery**	16.99 ± 11.12	18.35 ± 10.73	0.41^a^
**History of twin pregnancy**	2 (2.4)	4 (4.3)	0.48^b^
**History of abortion**	0.45 ± 0.80	0.25 ± 0.59	0.07^a^
**History of breastfeeding**	60 (72.3)	70 (76.1)	0.57^b^
**History of Infertility**	7 (8.4)	7 (7.7)	1^b^
**History of PCO**	4 (4.8)	2 (2.2)	0.34^b^
**History of OCP use**	25 (30.1)	26 (28.3)	0.79^b^
**History of HRT use**	6 (7.2)	5 (5.4)	0.52^b^

^a^ Independent Student t-test. ^b^ Chi-square test. *PCO* polycystic ovarian disease, *OCP* oral contraceptive, *HRT* hormone replacement therapy

### Size Changes in largest and Smallest Fibroadenomas

Table 2 compares fibroadenomas size changes between the two groups. When we analyzed the largest mass in each woman, the two groups were not statistically different before the intervention, and the largest mass size had significantly decreased after the intervention in both groups (Paired t-test, *p*-value < 0.05). However, for the smallest mass, a significant size reduction had only occurred in the treatment group. Table 2 shows the changing size of the largest mass was not a statistically significant difference between the two groups (2.04 ± 6.67 vs. 2.40 ± 4.96, *p*-value = 0.69). However, decreasing size in the smallest mass was statistically higher in the treatment group compared with the placebo group (3.26 ± 3.26 vs. 0.14 ± 6.48, *p*-value = 0.007). On the other hand, although a less than 20 % increasing the size of mass as a favorable outcome was higher in the treatment group, only in the smallest mass the differences between the two groups was statistically significant (*p*-value = 0.009).

**Table 2:** Comparison of Fibroadenomas size changes in the two groups

	Metformin Group	Placebo Group	p-value
**Average of largest mass size before intervention (mm)**	13.06 ± 4.46	13.52 ± 5.96	0.57
**Average of largest mass size after intervention (mm)**	10.66 ± 6.49	11.48 ± 8.11	0.47
**Change in size of the largest mass (mm)**	2.40 ± 4.96	2.04 ± 6.67	0.69
**Average of smallest mass size before intervention (mm)**	6.81 ± 2.25	7.65 ± 3.36	0.19
**Average of smallest mass size after intervention (mm)**	3.58 ± 4.21	7.51 ± 7.38	**0.006**
**Change in size of the smallest mass (mm)**	3.26 ± 3.26	0.14 ± 6.48	**0.007**
** A less than 20 % increase of largest mass, n (%)**	74 (94 %)	81 (88 %)	
**A more than 20 % increase of largest mass, n (%)**	5 (6 %)	11 (12 %)	0.17
** A less than 20 % increase of smallest mass, n (%)**	41 (95.3 %)	27 (75 %)	
**A more than 20 % increase of smallest mass, n (%)**	2 (4.7 %)	9 (25 %)	**0.009**

*P*-value refers to Chi-square and Student T-test, when appropriate

**Fig. 2:**
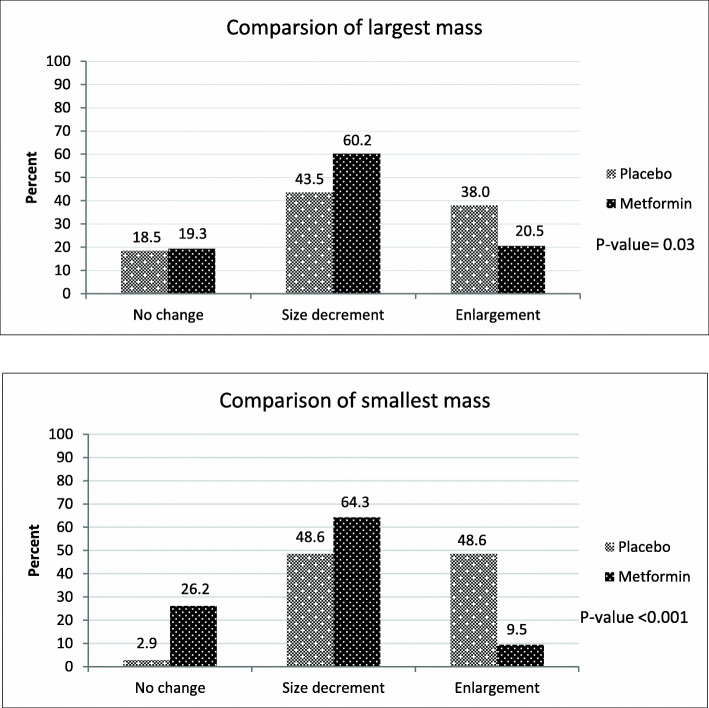
The percentage of fibroadenoma size changes after intervention in the largest (Up) and the smallest mass (Down) in each woman was shown by column chart

Figure 2 shows the FA size changes in the largest and smallest masses after the intervention considering three categories (no change, enlargement, size decrement). As it is shown in the chart, in the largest FAs, the rate of size reduction was higher in the treatment group (60.2 % vs. 43.5 %); while a higher rate of enlargement was observed in the placebo group (38 % vs. 20.5 %). In the smallest FAs, about 90 % of the masses got smaller or remained stable in the treatment group. However, about 50 % of the smallest mass in the placebo group had enlarged. Overall, a comparison of FA size changes between the treatment and placebo groups shows statistically significant differences in mass changes in both largest and smallest FAs (*p*-value < 0.05).

### Change in number of detectable lesions

In order to compare the rate of vanishing of FAs in the two groups, we used the mean number of FAs in each individual before and after the intervention. The average number of masses before the intervention was statistically similar in both groups (p-value = 0.07); however, after intervention with MF, the average number of masses had significantly decreased (2.39 ± 2.04 vs. 1.96 ± 2.32, p-value = 0.005).

Table 3 shows changes in the number of masses in all participants as well as in single and multiple fibroadenomas. In the multiple mass, the rate of FAs that had complete disappearance was higher in the treatment group compared with placebo (13.6 % vs. 8.3 %); and the rate of increased number was lower in the treatment group (9.1 % vs. 27.8 %). These differences in multiple mass show a borderline statistical significance (p-value = 0.08) between the two groups. However, changes in the number of mass in single mass cases were similar in the two groups.

**Table 3:** Changes in the number of masses in all participants and in single and multiple fibroadenomas

	**Metformin Group**	**Placebo Group **	***p*-value**
**Single mass-total**; n (%)	39 (100)	56 (100)	0.74
Disappearance	4 (10.2)	7 (12.5)	
Without change	34 (87.2)	46 (82.1)	
Increased number	1 (2.6)	3 (5.4)	
**Multiple mass-total**; n (%)	44 (100)	36 (100)	0.08
Disappearance	6 (13.6)	3 (8.3)	
Without change or decreased	34 (77.3)	23 (63.9)	
Increased number	4 (9.1)	10 (27.8)	
**In all cases**; n (%)	83 (100)	92 (100)	0.21
Disappearance	10 (12.1)	10 (10.9)	
Without change or decreased	68 (81.9)	69 (75)	
Increased number	5 (6)	13 (14.1)	

*P*-value refers to Chi-square test.

### Overall Change in Average Size of all Fibroadenomas

Overall, women in the placebo group had 172 FAs, and patients in the treatment group had 190 FAs at the point of entry in the study. In women with multiple masses, the logistic regression model showed that the odds for < 20 % enlargement in even one mass of a patient was more than four-fold in the treatment group in comparison with the placebo group (OR = 4.67, 95 % CI: 1.34–16.28, *p*-value = 0.02). However, this difference did not apply in patients with single FAs (*p*-value = 0.938).

The result of marginal model analysis when considering overall mass size as the response variable showed that the amount of size regression was more than two-fold in the treatment group compared with the placebo group after the intervention (30.57 % size reduction in the former vs. %14.1 size reduction in the latter group); this difference was statistically significant (*p*-value = 0.03).

The marginal logistic regression model showed an odds ratio (OR) of 1.48 [95 % confidence interval (CI): 1.10–1.99] for less than 20 % increase in a 6-month interval in the treatment group in comparison with the placebo group (*p*-value = 0.01).

### Drug adverse effects and patient compliance

In the treatment arm, all participants except one complied with the intervention and consumed the medicine according to the research protocol. Drug discontinuation happened in three women in the placebo group. Three of the women using MF reported some bloating during the first weeks of medicine consumption, and the symptom disappeared after a couple of weeks in all three. No serious adverse effect was seen secondary to MF consumption.

## Discussion

In this study, use of MF as a treatment for breast FA has been assessed for the first time, and some superior effect has been detected for MF compared to placebo.

Many treatments have been proposed for FA, comprising a spectrum from pure observation to surgical excision. Surgical excision is certainly the most effective treatment of FA. However, this is an invasive modality, and the objective of studies is to find the best non-invasive substitute. The high recurrence rate of vacuum-assisted excision of FA [12], the frequent conversion of endoscopic to open excision [13], and the invasive nature of these techniques exclude them from first-choice options. Non-surgical ablation techniques have a rate of complete shrinkage of around 70–80 % in different methods [14–17]. These rates are notable and exceed the disappearance rate caused by MF in our study. However, the effects appear very gradually during around one year, while MF was prescribed for only six months in our study. Also, the dose of MF was low, while doses around 1500 to 2000 mg are used for other purposes. Therefore, longer usage or a higher dose could lead to similar or superior results. In addition, ablation methods are minimally invasive and rely on access to advanced equipment; this shifts the advantages toward MF, which can be easily available and used everywhere.

Several clinical studies and reviews had proposed a conservative approach to FA as soon as the 1980 and 1990 s; in favor of only observing and following up the size of cytologically- or histologically-proved small FAs in women who opt for it, are younger than 35 years of age, and have no family history or other risk factors for breast cancer [18, 19]. This approach is acceptable, but when FA size or symptoms dictates treatment, or the patients are unwilling to undergo the “watch and wait” mode, an effective medical treatment could be the best conservative management.

Various medications have been explored in this regard. Evening primrose oil is rich in gamma-linolenic acid, which is known to affect the metabolism of prostaglandins and has thus been investigated for treatment of benign breast conditions [20]. Kollias et al. [21] assessed its effects on FAs smaller than 3 cm, and half of their 21 cases got smaller, with no significant difference with the control group. This is in contrast with MF, which showed significantly better effects in FA treatment than the placebo.

Considering the estrogenic-dependent features of FA, anti-estrogenic compounds have been brought to trials that dealt with FA. Tamoxifen is a selective estrogen receptor modulator (SERM) widely used in breast cancer treatment and prevention that has seldom been studied for treatment of benign diseases of the breast, but a decrease in risk of developing FA [22], and a size reduction of existing FA [23] have been shown to be induced by tamoxifen; however the rate of shrinkage has not been explored. Centchroman is another SERM that has been prescribed for a period of 3 or 6 months for treatment of FA; the masses completely disappeared in 28–44 % and showed size reduction in around 30 % [7, 24–26]. Although the rate of disappearance of FA is higher than the rates in our study (28–44 % vs. 12.1 %) (Table 3), the rate of FA size reduction is higher in our study and more than 60 % of the largest and the smallest mass in treatment group had size reduction (Fig. 2). On the other hand, SERMs have several bothering side effects, including hot flushes, menstrual irregularity, headache, depression, thromboembolic events, ocular disturbances, leg cramps, endometrial hyperplasia, uterine polyps, and endometrial cancer [27, 28]; these side effects prohibit their widespread consumption for management of benign disorders. Contrarily, MF is a medicine with an approved safety profile and tolerability [29]. The most common adverse effects of MF consist of gastrointestinal disturbances such as mild anorexia, diarrhea, nausea and vomiting, or abdominal discomfort. Other adverse effects are more serious but very rare and include lactic acidosis, hepatotoxicity, acute pancreatitis, pernicious anemia, or hypoglycemia with high doses of MF (e.g. 850 mg × 3 daily) [30]. In our study, no serious adverse effect was recorded, and the medicine was well-tolerated by all users. Also, the compliance of women in consuming the medicines was excellent in the treatment group. Therefore, the much lower rate of serious adverse effects of MF, and the high rate of compliance in comparison with SERMs [31, 32] make use of MF probably more applicable.

MF has been investigated in many studies regarding its probable anti-cancer properties, including breast cancer. Several mechanisms of action have been suggested and explored. In vitro studies have shown that MF has anti-proliferative and growth inhibitory effects. These are mediated through various mechanisms, comprising inhibition of synthesis of fatty acids, activation of AMP-activated protein kinase, reduction of mitochondrial metabolites, amplified apoptosis, reduced colony formation, cell cycle arrest at the G1 checkpoint, inhibition of mTOR and decreased expression of E2F1 and cyclin D1 [33–35]. The decrease of cellular division by MF is seen in both estrogen positive and negative breast cancer [34, 36] MF has been shown to reduce both estrogen-dependent and basal breast cancer cell proliferation [37].

Our rationale for anticipating a positive role for FA was related to the anti-proliferative actions described for MF in breast cancer cells, and the pathophysiology of FA formation, since FA consists of stromal changes including cellularity, and variants of proliferative epithelial changes [3, 4]. Also, MF has anti-estrogenic features which have made it an effective adjunct in management of some ovarian function and reproductive disorders, such as polycystic ovarian disease and estrogen-dependent infertility [38, 39]. This also is a suggestion for use of MF in other sex-hormone-related disorders, like FA.

To our knowledge, this is the first study investigating the benefit of MF in the management of FA. Considering the importance of size stability of FA in clinical practice, we considered a size change of less than 20 % enlargement as a success; in contrast a 20 % or more enlargement of FA was regarded as a failure [8–10]. Therefore, according to the marginal logistic regression model, our study showed an OR of 1.48 for success in the treatment group. Also, by defining success as less than 20 % FA enlargement in all the masses of a patient with multiple lesions, our study illustrated a more than four-fold success rate for metformin in these cases.

In summary, according to our expectation, MF showed a favorable effect on FA in our study. The most important positive findings can be briefly interpreted as: (1) Significant enlargement was less probable in FAs under MF treatment. (2) In women with multiple FAs, MF increased more than four-fold the probability of a safe course for all the masses of a patient by decreasing the chance for significant enlargement. (3) FAs under MF treatment had a two-fold size decrement compared with those under placebo. (4) For the largest FA of each patient, size reduction was more frequent and enlargement was less frequent with MF than with placebo. (5) Women receiving MF had experienced a two-fold regression of FA size in comparison with the placebo group in all fibroadenoma. (6) The rate of disappearance of FAs was higher in largest and smallest fibroadenoma under MF treatment in women with multiple lesions.

The number of the largest FAs which had disappeared was not statistically different in the two groups; these could show statistically significant in a larger sample size. Also, the dosage of MF that was prescribed in this study was only 1000 mg daily, while many studies prescribe 1500–2000 mg of MF per day. Prescribing a higher dose of MF could probably improve the positive findings of the study.

Other than the present study, MF has not yet been investigated as a medical treatment of FA; but as far as we know, the effect of MF on benign breast lesions has been reported in one published study. Talaei et al. have compared the effects of MF, a placebo, and no treatment in women with fibrocystic breast changes. They detected a significant improvement in cysts number and size as well as breast tenderness and nipple discharge in the MF group in comparison with the two other groups [40].

Our study had some limitations. We did not measure the glucose tolerance and serum insulin levels, or blood sex hormone levels, which could have been useful in interpreting possible treatment effects. The medium size of FAs was small in our study because we did not define any minimum size limit as inclusion criteria. Another limitation of our study was the inclusion of women with both single and multiple FAs, which might behave differently clinically; and we had no method to prove that all the small ultrasound findings that mimicked FAs were true FAs. Also, we could not follow around 17 % of cases due to COVID-19 limitations that restricted hospital access for non-emergent and non-malignant cases. Since our favored result was to observe a lower rate of mass enlargement in the treatment group; considering the rate of increased mass size in Fig. 2, the calculated power (using Epi Info Site) in the largest and smallest mass was 71.93 and 99.99 %, respectively.

## Conclusions

In conclusion, this is the first study which evaluates the effect of MF as a management option in breast FA. The results suggest a favorable effect, especially in women with multiple FAs. Furthermore, the effect of MF was obviously significant especially in small masses. Larger studies using higher doses of MF and including a separate design for patients with single or multiple FAs are suggested in order to confirm this effect.

## Data Availability

All data analyzed during this study are included in this published article and row-data are available from the corresponding author on reasonable request.

## Abbreviations

FA: Fibroadenoma
OR: Odds ratio
US: Ultrasound
MF: Metformin
IRCT: Iranian Registry of Clinical Trials
BMI: Body mass index
GEE: Generalized estimate equation
CI: Confidence interval
SERM: Selective estrogen receptor modulator
